# Transition Metal Oxide Electrode Materials for Supercapacitors: A Review of Recent Developments

**DOI:** 10.3390/nano11051248

**Published:** 2021-05-10

**Authors:** Ruibin Liang, Yongquan Du, Peng Xiao, Junyang Cheng, Shengjin Yuan, Yonglong Chen, Jian Yuan, Jianwen Chen

**Affiliations:** 1 School of Physics and Optoelectronic Engineering, Foshan University, Foshan 528000, China; leungseoiban@163.com (R.L.); du_0258@163.com (Y.D.); cheng-jy@outlook.com (J.C.); ysj981006@163.com (S.Y.); c19875916364@163.com (Y.C.); yuanjian054@163.com (J.Y.); 2 Guangdong-Hong Kong-Macao Joint Laboratory for Intelligent Micro-Nano Optoelectronic Technology, Foshan University, Foshan 528225, China; 3 School of Electronic and Information Engineering, Foshan University, Foshan 528000, China; iamjwen@126.com

**Keywords:** energy storage devices, supercapacitors, transition metal oxides, developing trend

## Abstract

In the past decades, the energy consumption of nonrenewable fossil fuels has been increasing, which severely threatens human life. Thus, it is very urgent to develop renewable and reliable energy storage devices with features of environmental harmlessness and low cost. High power density, excellent cycle stability, and a fast charge/discharge process make supercapacitors a promising energy device. However, the energy density of supercapacitors is still less than that of ordinary batteries. As is known to all, the electrochemical performance of supercapacitors is largely dependent on electrode materials. In this review, we firstly introduced six typical transition metal oxides (TMOs) for supercapacitor electrodes, including RuO_2_, Co_3_O_4_, MnO_2_, ZnO, XCo_2_O_4_ (X = Mn, Cu, Ni), and AMoO_4_ (A = Co, Mn, Ni, Zn). Secondly, the problems of these TMOs in practical application are presented and the corresponding feasible solutions are clarified. Then, we summarize the latest developments of the six TMOs for supercapacitor electrodes. Finally, we discuss the developing trend of supercapacitors and give some recommendations for the future of supercapacitors.

## 1. Introduction

Owing to the rapid development of the global economy, accelerating the consumption of fossil fuels like coal, fuel, and natural gas, the problems of global climate change and environmental pollution are increasing to significant levels. Developing solar, wind, tidal, and other renewable clean energy is a way to mitigate current energy and environmental pollution problems [1,2,3,4,5,6]. However, those renewable clean forms of energy are severely restricted by environmental factors and energy supply is intermittent [7]. Therefore, it is urgent to develop effective and reliable devices for energy storage [8,9]. As a new type of storage device, supercapacitors have gained great attention in recent years thanks to their advantages of fast charge/discharge rate, high power density, and very long cycle life [10,11,12,13,14]. Supercapacitors have great potential in the area of portable electronic equipment, renewable energy systems, and hybrid power cars [15,16,17]. There are two categories of supercapacitors: electrical double-layer capacitors (EDLCs) and pseudocapacitors [18,19]. For EDLCs, energy is stored with the charge accumulating on the interface between the electrode and electrolyte. For pseudocapacitors, the energy is stored through a fast and reversible faradaic redox reaction [20,21,22]. At present, the low energy density is still the bottleneck of supercapacitors [23,24]. According to the energy density formula, $E=\frac{1}{2}\times C\times V^{2}$, where *E* represents the energy density, *C* represents the specific capacitance, and *V* represents the potential window, we can know that the energy density of a supercapacitor depends on both capacitance and operating voltage, and it can be enhanced by increasing the potential window and using an electrode material with high capacitance [25,26]. It is noteworthy that the design and preparation of electrode materials are the key steps to determine the electrochemical performance of supercapacitors. In other words, the performance of supercapacitors is highly dependent on electrode materials [27,28,29,30].

Electrode materials can be divided into three categories: carbon materials, conductive polymers, and transition metal oxides (TMOs). Carbon materials are usually used as electrode materials for EDLCs. They have the characteristics of high specific surface area, adjustable pore size distribution, and excellent electrical conductivity [31,32]. Owing to the storage mechanism of EDLCs, carbon materials can provide high power density, but low energy density, which limits the overall performance [33,34]. Carbon materials such as graphene, carbon nanotubes, and carbon nanofibers have been widely studied for electrode materials. However, low specific capacitance of carbon material limits the capacity of the EDLCs [35], and the applications of those carbon materials are limited by their high cost [36]. The conductive and pseudocapacitive properties of conductive polymers are good, but the stability is poor, and conductive polymers can easily fall off the substrate [37,38]. For example, the charge and discharge speed of polyaniline is low and the stability of the charge/discharge process is poor [39,40]. TMOs have higher specific capacitance (100–2000 F g^−1^), higher energy density than carbon materials, and better chemical stability than conductive polymers [41,42,43]. Thanks to its high theoretical capacitance and rapid faraday redox reaction, RuO_2_ is thought to be an optimal pseudocapacitive electrode material [44,45,46]. However, its high price and toxicity to the environment seriously hinder its application in supercapacitors [47,48,49]. Co_3_O_4_, MnO_2_, and ZnO have the advantages of being natural abundant and high specific capacitance, which makes them substitutes for RuO_2_ [50,51]. The disadvantage of poor electrical conductivity is exhibited by many metal oxide electrodes [52]. Because of the co-existence of two metal ions and synergistic effects of elements, ternary metal oxides, AB_2_O_4_ (A or B = Ni, Co, Mo, Mn, and so on), have more active reaction sites and high electrical conductivity than binary metal oxides [53]. In addition, spinel cobaltates (XCo_2_O_4_, X = Ni, Cu, Zn, Mn, and so on) and metal molybdate (AMoO_4_, A = Ni, Mn, Co, and so on) have received tremendous research interest because of their low cost, enhanced electrochemical activity, and being a natural abundant resource [54,55].

In this review, six transition metal oxide materials including RuO_2_, Co_3_O_4_, MnO_2_, ZnO, XCo_2_O_4_ (X = Mn, Cu, Ni), and AMoO_4_ (A = Co, Mn, Ni, Zn) are firstly introduced. Secondly, existing problems of those TMOs in practical application and the corresponding methods are presented. The methods include synthesizing composites, preparing material in nano scale, introducing battery-type material or oxygen vacancies, and modification of quantum dots. Particularly, we introduce the latest developments of six TMOs for supercapacitor electrode according to the strategies. Finally, the future development of supercapacitors is discussed and recommendations on supercapacitors are exhibited.

## 2. TMOs-Based Electrode Materials

### 2.1. RuO_2_

RuO_2_ has high theoretical capacitance (2000 F g^−1^) and it can carry out rapid faraday redox reaction. It has good electrical conductivity, high chemical and thermal stability, as well as a big voltage window. Thus, RuO_2_ is called the most ideal pseudocapacitive electrode material [40,56]. However, ruthenium is too expensive to be used for large-scale synthesis of electrode materials.

#### 2.1.1. RuO_2_-Based Composite Electrodes

**Figure 1:**
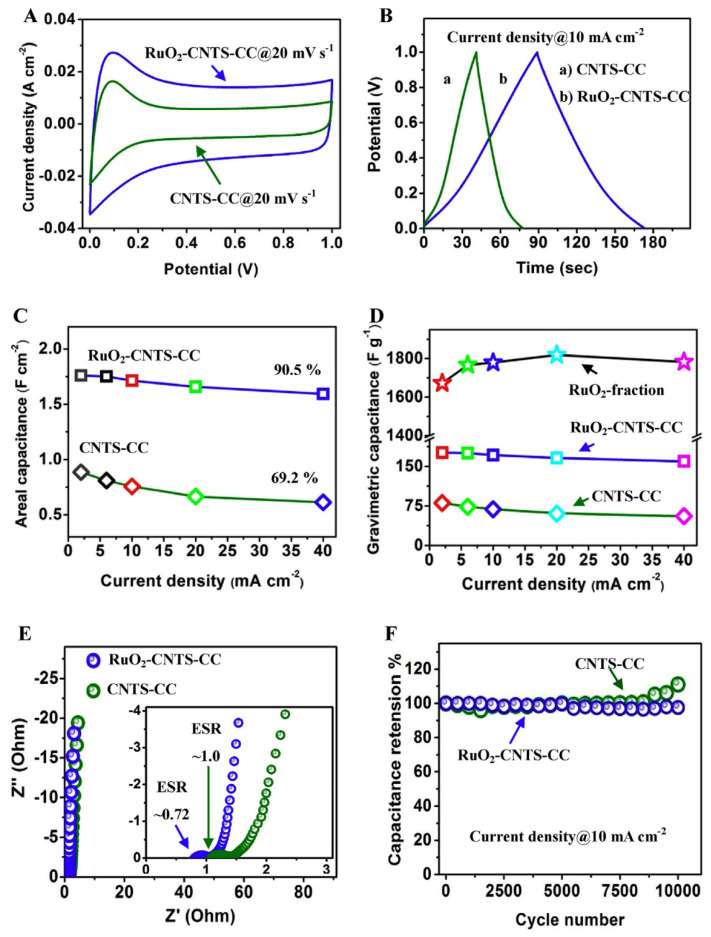
Electrochemical performance of different electrodes in liquid state supercapacitors using 1 M H_2_SO_4_ electrolytes: (**A**) comparison of CV at 20 mV s^−1^; (**B**) GCD curves at 10 mA cm^2^; (**C**) calculated areal capacitance at a range of current densities; (**D**) calculated gravimetric capacitance at different current densities; (**E**) Nyquist plots of different samples, and the inset showing the corresponding magnified high-frequency regions; and (**F**) cyclic stability over 10,000 cycles at 10 mA cm^−2^. Reproduced with permission from [62]. Copyright 2019 Elsevier. CNTS-CC, carbon nanotubes grown carbon cloth. CV, cyclic voltammetry. GCD, galvanostatic charge-discharge. ESR, equivalent series resistance.

Synthesizing composites can improve the utilization of RuO_2_ and reduce the cost of RuO_2_ electrode [57]. At the same time, the synergistic effect of the materials is beneficial to improve the electrochemical performance of the electrode. Various forms of RuO_2_-based electrodes have been prepared such as metal sulfide-RuO_2_ electrode, metal oxide-RuO_2_ electrode, carbon material-RuO_2_ electrode, and multicomponent RuO_2_-based electrode [39,57,58,59,60,61]. Asim et al. [62] decorated carbon nanotubes (CNTs) grown carbon cloth (CNTS-CC) with RuO_2_ nanorods (RuO_2_-NRs) via chemical vapor deposition (CVD) and the annealing process. The prepared electrode has the characteristics of both supercapacitors and lithium batteries. The results show that the designed material has high specific capacitance (176 F g^−1^) and superior cycling stability (97% retention after 10,000 cycles at 40 mA cm^−2^) (Figure 1).

In addition, they demonstrate a reversible capacity of ~3.85 mAh cm^−2^ at the current density of 100 mA cm^−2^ as a Li-ion batteries electrode. The excellent electrochemical performance lies in the synergistic effect of metal oxide NRs and the unique structure of CNTS-CC. Enlarged surface area and exposed active sites allow accessibility of the ions/electrolytes to the electrodes. Ternary single-walled carbon nanotubes/RuO_2_/polyindole (SWCNT/RuO_2_/PIn) nanocomposite fabricated by Zhu’s group [63] presents the specific capacitance of 1283 F g^−1^ at 1.0 A g^−1^. At 500 W kg^−1^ and 5000 W kg^−1^, the energy densities of the SWCNT/RuO_2_/PIn electrode-based symmetric supercapacitor are 42 W h kg^−1^ and 33 W h kg^−1^, respectively. It also shows good cycling stability and capacitive performance of 1203 F g^−1^ at 1.0 A g^−1^. Via the hydrothermal and annealing process, CuCo_2_O_4_/CuO nanoneedles were synthesized directly on the conductive Ni foam (NF), on which RuO_2_ nanoparticles were deposited [64]. The CuCo_2_O_4_/CuO@RuO_2_ can work as water oxidation catalysis and has low overpotential (279 mV at 10 mA cm^−2^), low Tafel slope, and stable long-term performance. Besides, it achieves a high areal capacity up to 862.5 mAh cm^−2^ and a capacity retention of about 90.1% after 8000 cycles. The hybrid supercapacitor fabricated by CuCo_2_O_4_/CuO@RuO_2_ and activated carbon achieves an energy density of 0.84 mW h cm^−2^ at 8 mW cm^−2^.

#### 2.1.2. Hydrographic RuO_2_

**Figure 2:**
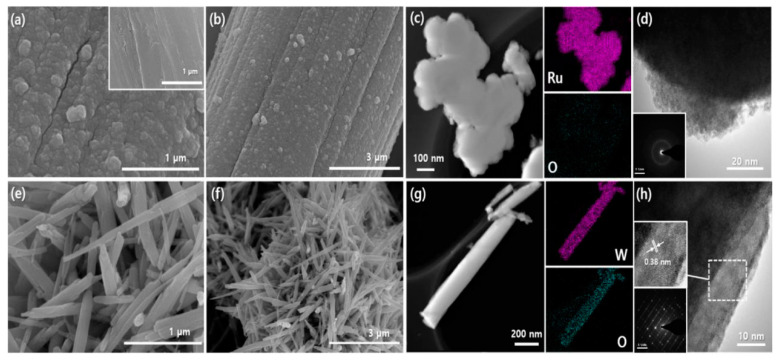
(**a**,**b**) Inset of (**a**) FE-SEM images of hydrous RuO_2_ on carbon cloth at different magnifications; (**c**) TEM image of the hydrous RuO_2_ and its EDS mapping; (**d**) HRTEM image of hydrous RuO_2_ and its SAED pattern in the inset of (**d**); (**e**,**f**) FE-SEM images of h-WO_3_ on carbon cloth at different magnifications; (**g**) TEM image of the h-WO_3_ nanorod and its EDS mapping; (**h**) HRTEM image of h-WO_3_ nanorod. The inset shows the SAED pattern, reproduced with permission from [72] Copyright 2019 Elsevier. FE-SEM, field emission scanning electron microscopy. TEM, transmission electron microscope. EDS, energy dispersive X-ray spectroscopy. HRTEM, high resolution transmission electron microscope. SAED, selected area electron diffraction.

In the reports above, many electrode materials have the addition of carbon materials. Theoretically, adding carbon materials can improve the surface area and conductivity of electrodes [65]. However, some reports pointed out that the porous surface of carbon materials was blocked by RuO_2_ particles, which results in reduced surface area, limited electrochemical properties, and decreased double layer capacitance [66,67]. Hydrated RuO_2_ is a kind of nanocrystalline material with RuO_2_ as the core and a small amount of water hydrogen bond on the surface. The form of porous structure provides conduction paths for protons to enter the inner part of RuO_2_ [68,69]. The annealing treatment of activated carbon-RuO_x_•nH_2_O composites (annealing for 2 h in 200 °C air) obviously improves the discontinuity of the electron path in particles. Further, the obstacle of electron hopping between particles is reduced because a large amount of active carbon (AC) powder is introduced. The specific capacitance of the RuO_x_•nH_2_O is 1340 F g^−1^ (at 25 mV s^−1^) [70]. Similarly, Yang’s group [71] synthesized RuO_2_@SWCNTs/graphene (S/G) composites by the sol–gel method. The hybrid electrode material, RuO_2_@S/G-60, has a specific capacitance up to 988 F g^−1^ and excellent rate capability at 1 A g^−1^. Hexagon WO_3_ (h-WO_3_) and hydrous RuO_2_ were respectively prepared on three-dimensional conducting carbon cloth (CC) via the hydrothermal method [72], which meant fast electronic pathway, high conductivity substrate, high surface area, and enhanced electrochemical performance. Figure 2a–d shows the field emission scanning electron microscopy (FE-SEM) images, transmission electron microscope (TEM) image, and high resolution transmission electron microscope (HRTEM) image of hydrous RuO_2_ at different magnifications. Figure 2e–h shows the FE-SEM images, TEM image, and HRTEM image of h-WO_3_ at different magnifications.

The asymmetric supercapacitor was fabricated in H_2_SO_4_ aqueous electrolyte with h-WO_3_/CC as negative electrode and hydrated RuO_2_/CC as positive electrode. At an operating voltage of 1.6 V, the asymmetric supercapacitor device is optimized with volumetric capacitance of 3.52 F cm^−3^ at 5 mA cm^−2^. Moreover, at a power density of 40 W cm^−3^, the asymmetric supercapacitor device shows brilliant energy density of 1.25 W h cm^−3^ and it also shows promising electrochemical stability. By comparison, an all-pseudocapacitive asymmetric device [73] has an energy density of only 37 µW h cm^−2^ at 40 mW cm^−2^, but a remarkable 96% retention after 20,000 charge/discharge cycles.

### 2.2. Co_3_O_4_

Co_3_O_4_ is a kind of transition metal oxide belonging to the spinel family, and the faradic redox reaction of Co_3_O_4_ can be described as follows [74]:

(1) $${\mathrm{Co}}_{3}O_{4}+{\mathrm{OH}}^{-}↔3\mathrm{CoOOH}+e^{-}$$

(2) $$\mathrm{CoOOH}+{\mathrm{OH}}^{-}↔{\mathrm{CoO}}_{2}+H_{2}O+e^{-}$$

Co_3_O_4_ has the advantages of high theoretical specific capacitance (3560 F g^−1^), low price, environmental friendliness, and good chemical durability, so it is a promising active material [75,76,77]. However, the capacitance in practical application differs a lot from the theoretical value. A reason is that electron transfer is hindered owing to low conductivity, slow kinetics, large volume expansion-contraction, and severe particle aggregation. Thus, the capacitance and cycling performance of Co_3_O_4_ are limited [78,79].

#### 2.2.1. Co_3_O_4_ Nanomaterials

**Figure 3:**
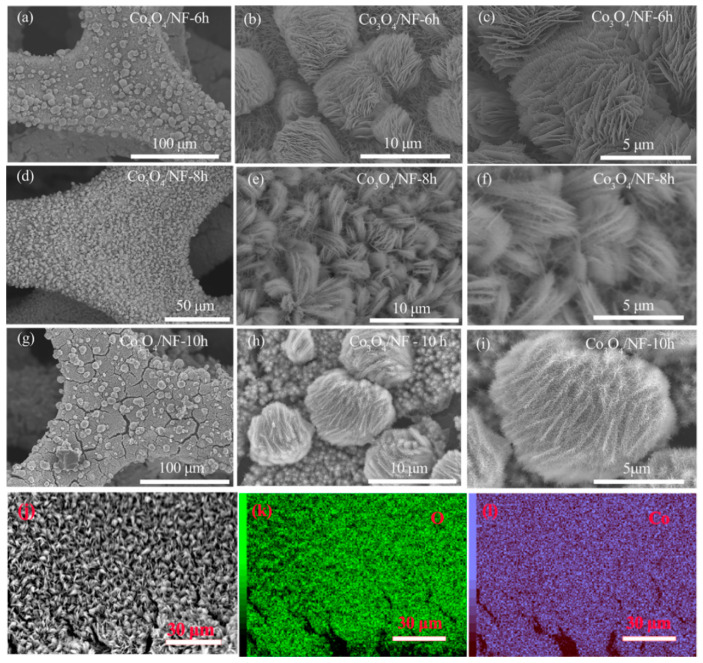
SEM images: (**a**–**c**) Co_3_O_4_/nickel foam (NF)—6 h; (**d**–**f**) Co_3_O_4_/NF—8 h; (**g**–**i**) Co_3_O_4_/NF—10 h; (**j**–**l**) element mapping images of Co_3_O_4_ nanoflakes, reproduced with permission from [93] Copyright 2021 Elsevier. NF, Ni foam.

Nanomaterials have a large surface with numerous active sites. The ultra-small size of nanomaterials shortens the electron/ion diffusion path, providing favorable transfer pathways [78]. Because the dimension and morphology of the materials play an important role in improving the electrochemical performance, increasingly more attention has been paid to the synthetic materials in micro and nano scale [80]. A variety of Co_3_O_4_ nanomaterials have been synthesized, such as nanowires [81], nanofibers [82], nanoparticles [83,84], and nanosheets [85]. Yue and co-workers [86] used a simple and environmentally friendly one-step hydrothermal and calcination method to prepare Co_3_O_4_/reduced graphene oxide (rGO) composites. Tiny Co_3_O_4_ nanoparticles are dispersed on rGO flakes and a three-dimensional structure is formed. The prepared electrode has more active sites than Co_3_O_4_ nanowires [87] to achieve better electrochemical performance. The optimized electrode Co_3_O_4_/rGO-120-12 shows high specific capacitance of 1152 F g^−1^ at 1 A g^−1^. Indira Priyadharsini [88] firstly prepared Co_3_O_4_ nanoparticles by the sol–gel method. The obtained Co_3_O_4_ nanoparticles, carbon, and polyvinylidene fluoride (PVDF) were then mixed with a mass ratio of 8:1:1. The electrode was prepared by coating the synthesized slurry uniformly on the Ni foam substrate. The specific capacitance of the electrode at 11 mA cm^−2^ is 761.25 F g^−1^. The reason for the low specific capacitance is that the addition of PVDF increases the resistance between the collector and the redox active material, which results in a decrease in the number of active sites and the electron/charge transport rate [89]. However, directly growing Co_3_O_4_ active materials on conductive substrates such as carbon fiber, carbon cloth, stainless steel sheet, and metal foam can avoid the addition of binder and conductive additive [51]. Ag doped Co_3_O_4_ nanosheets synthesized on Ni foam [90] possess a specific surface area (176 m^2^ g^−1^) larger than the original Co_3_O_4_ (108 m^2^ g^−1^). There is still a superior specific capacitance of 1323 F g^−1^ at the current density of 10 A g^−1^ (92.84% of the initial specific capacitance), indicating outstanding rate capability of the sample. Furthermore, it exhibits 104.7% of the initial capacitance at the first 2000 cycles. The electrode materials prepared by Yang’s group [91] and Wang et al. [92] both show good electrochemical properties (883 F g^−1^ and 1606.6 F g^−1^ at the current density of 1 A g^−1^, respectively). Electrode prepared by Wei [93] has unique nanoarray structures. The SEM images of Co_3_O_4_/NF with different heating times are shown in Figure 3a–i. Figure 3j–l shows the element mapping images of Co_3_O_4_ nanoflakes.

Co_3_O_4_ nanowires were anchored on the surface of the Co_3_O_4_ nanosheet, effectively expanding the surface area, providing rich active states for the faradaic redox reaction and promoting the diffusion rate of electrolyte ions. The sample prepared at hydrothermal heating for 8 h (Co_3_O_4_/NF-8 h) shows a high specific capacitance of 2053.1 F g^−1^ at 1 A g^−1^. The working voltage of the hybrid supercapacitor device assembled by the hetero-structured Co_3_O_4_ array and graphene can reach 1.6 V. The maximum energy density of 22.2 W h kg^−1^ is delivered and the excellent cycle is proved by the 93.3% capacitance retention after 10,000 cycles.

#### 2.2.2. A Strategy for Preparation of Co_3_O_4_ Nanomaterials

In recent years, metal organic frameworks (MOFs)-based materials have attracted attention in various fields of gas adsorption, energy device, pollutant degradation, electrocatalysis, and drug release [1]. Besides, with adjustable porous structure, controllable pore size distribution, and high surface area, MOFs are thought to be an ideal template for the preparation of Co_3_O_4_ nanomaterials [94,95]. Commonly, Zeolitic Imidazolate Frameworks-67 (ZIF-67) is used as precursor to synthesize Co_3_O_4_ nanomaterials. With ZIF-67 crystals as precursor, porous Co_3_O_4_ nanoparticles [96] were transformed by simple calcination treatment. The results show that ZIF-67-derived Co_3_O_4_ nanoparticles exhibit high specific capacitance (190 F g^−1^ at 5 A g^−1^) and capacitance retention (71.42% after 5000 cycles). Bao and co-workers [97] used ZIF-67 as a template and successfully constructed a series of Co_3_O_4_ embedded α-Co/Ni(OH)_2_ hollow nanocages. The prepared composite (α-Co/Ni(OH)_2_@Co_3_O_4_-70) has abundant electrolyte diffusion channels and reactive active sites. At the same time, it possesses excellent conductivity and stability. Therefore, the high capacitance of 1000 F g^−1^ is obtained at 1 A g^−1^ and the capacitance retention is 74% at 10 A g^−1^. Meanwhile, the hybrid nanocages still maintain 72.34% of original capacitance after 8000 charge/discharge cycles. In addition, α-Co/Ni(OH)_2_@Co_3_O_4_-70//active carbon (AC) device has an energy density of 23.88 W h kg^−1^ at a power density of 0.075 kW kg^−1^. Wei et al. [98] developed a novel method (bottom-up approach) to synthesize ZIF-67 nanosheets. The ZIF-67 is in situ converted into Co_3_O_4_ ultrathin nanoparticles by thermal treatment. The energy density at 790.7 W kg^−1^ of asymmetric supercapacitors prepared with Co_3_O_4_//AC is 46.5 W h kg^−1^. Moreover, the overpotential of 2D Co_3_O_4_ ultrathin nanoparticles at the onset potential is 230 mV and Tafel slope is 74.0 mV dec^−1^. This shows 2D Co_3_O_4_ ultrathin nanoparticles’ great performance in oxygen evolution reaction. Besides, Co-based MOFs (Co-MOFs) have become fascinating templates for thermal transformation to generate porous Co_3_O_4_ structures [99]. Li et al. [100] prepared 3D porous carbon (3DPC)/Co_3_O_4_ composites by pyrolysis of 3D graphene/Co-MOF precursor. The electrochemical performance of the composites is not good (specific capacitance of 423 F g^−1^ at 1 A g^−1^ and capacitance attenuation of about 17% after 2000 cycles). This is because MOFs-based electrodes are usually composed of insulation binder, conductivity additives, and MOFs, which hinders the electrolyte penetration and reduces the electron transport between electroactive sites of active material and electrolytes [101].

#### 2.2.3. Electrode Materials on Conductive Substrates

**Figure 4:**
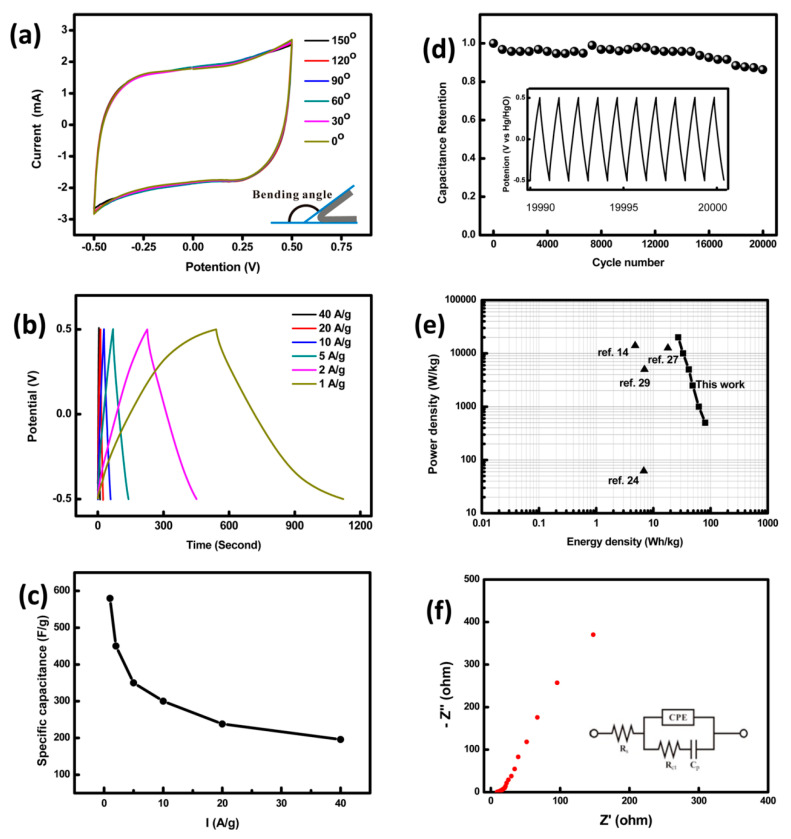
(**a**) CVs curve of the device at different bending angles; (**b**) GCD curves of the supercapacitor device at different current densities; (**c**) specific capacitance vs. current densities for the supercapacitor device; (**d**) cycling stability of the device at 20 A g^−1^. The inset shows the GCD curves of the last 10 cycles; (**e**) Ragone plots of the supercapacitor device in comparison with other reported supercapacitors; (**f**) Nyquist plot of the supercapacitor device, the inset is the equivalent circuit, reproduced with permission from [77]. Copyright 2015 ACS.

Synthesizing electrode materials directly on conductive substrates can avoid the addition of insulation binder so as to reduce interparticle resistance [51]. In order to further obtain high performance electrode materials, Han et al. [102] synthesized petal-like Co_3_O_4_@CoNi_2_S_4_ nano-wall arrays on carbon cloth. The synergistic effect of Co-MOF-derived Co_3_O_4_ skeleton is utilized to improve the electrochemical activity of cobalt nickel sulfide. The specific capacitance of the Co_3_O_4_@CoNi_2_S_4_ electrode is 244.4 mAh g^−1^ at 1A g^−1^ and a specific capacitance attenuation of 18.7% at 16 A g^−1^. At a power density of 884.4 W kg^−1^, the energy density of the Co_3_O_4_@CoNi_2_S_4_//AC device reaches 55.6 W h kg^−1^. After 10,000 cycles, the capacitance retention of Co_3_O_4_@CoNi_2_S_4_ reaches 86%. Liao and co-workers [77] prepared Co_3_O_4_ nanoparticles on graphene nanosheets supported by carbon fabric. The electrode shows an unbelievable specific capacitance of 3480 F g^−1^, which is close to the theoretical value (3560 F g^−1^). Besides, they fabricated a flexible symmetric supercapacitor device with two pieces of prepared electrode. The flexible supercapacitor has no sacrifices of electrochemical performance when it is bent under bending angles from 0° to 150°. Furthermore, the supercapacitor delivers capacitance of 580 F g^−1^ and 13.8% capacitance decline after 20,000 cycles. The maximum energy density of the device is 80 W h kg^−1^ (Figure 4).

### 2.3. MnO_2_

Owing to its rich natural content, no environmental pollution, and high theoretical specific capacitance (1380 F g^−1^), MnO_2_ has been widely studied as the most competitive transition metal oxide [103,104,105]. However, its application in supercapacitors is seriously affected because of its poor conductivity and slow ion transport rate [106,107,108].

#### 2.3.1. Carbon Materials@MnO_2_ Composite Electrode

**Figure 5:**
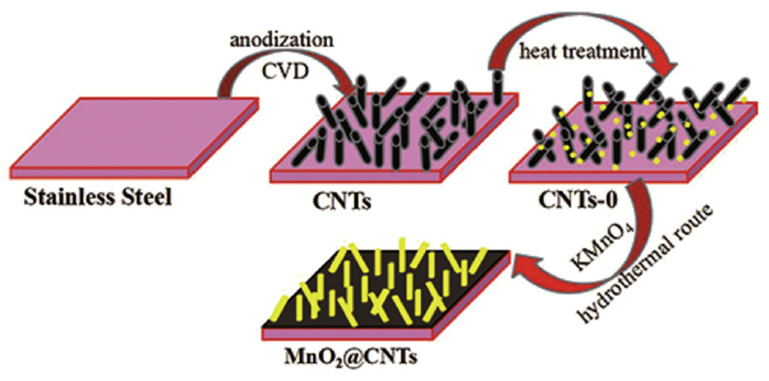
Schematic fabrication of core–shell MnO_2_@CNTs composite electrodes on stainless steel. Reproduced with permission from [119]. Copyright 2020 Elsevier.

Carbonaceous materials have excellent electrical conductivity and stability with large surface area [31,32]. Carbon nanotubes [109,110], graphene [111,112], carbon nanowires [113,114], carbon nanofibers [115], and other carbon materials can be used as scaffolds for depositing MnO_2_ nanostructures. Large surface area with a great quantity of active sites is provided and the contact path between electrode materials and electrolytes is shortened, which enhances electrochemical properties [116]. The hollow N-doped carbon (HNC)@MnO_2_ 3D core–shell composites prepared by Cai et al. [117] exhibit a good electrochemical capacity of 247.9 F g^−1^ at 0.5 A g^−1^, as well as a retention capacity of 82.9% at 5 A g^−1^ after 2000 cycles. Via a simple one-step water bath at 40 °C, Long et al. [118] developed interconnected δ-MnO_2_ nanosheets flexible electrode on activated carbon cloth. The electrode displays brilliant charge storage performance (a neglected degradation during the 2000 times of folding). When the power density of the asymmetric supercapacitor is 1198.4 W kg^−1^, the energy density can reach 49.8 W h kg^−1^. After 5000 cycles, the capacitance retention of the asymmetric supercapacitor can reach 90.6%. Lei and co-workers [119] synthesized MnO_2_ nanosheets@graphenated CNTs network on 316 L stainless steel by the CVD method followed by annealing treatment (Figure 5).

Providing an effective electronic pathway, graphenated CNTs are interconnected to form porous structures, with MnO_2_ nanoparticles uniformly distributed on the surface. Furthermore, the unique structure of MnO_2_ nanosheets@graphenated CNTs nanocomposites shortens the ion transport distance, promotes charge transfer, and accelerates reversible redox reactions, thereby improving the capacitance performance. A high energy density of 51.2 W h kg^−1^ is obtained at 24.42 W h cm^−2^ and the maximum power density is 0.4 kW kg^−1^ (200 W cm^−2^). 3D-graphene/MnO_2_ foam composite [120] was prepared by a combination of the CVD and hydrothermal method. The study shows that 3D-graphene/MnO_2_ composite electrodes in the absence of carbon black have a high specific capacitance of 333.4 F g^−1^ at 0.2 A g^−1^ and excellent cycling stability.

However, the high cost and complex preparation process of commercial carbon materials are not conducive to large-scale preparation. Therefore, it is very important to develop low price and renewable materials to meet the increasing demand. Biomass resource is a renewable resource with high utilization value. Biomass resource utilization has become a hot area and pillar of economic and social development in the future [121]. Biomass-derived carbon has the advantages of high natural abundance, environmental friendliness, diverse structures, long cycle life, and low costs [36,122,123]. In addition, the multistage pore structure and the inherent stability of carbon materials give biomass carbon unique advantages and prospects in the application of electrochemical materials. Hence, biomass-derived carbon has great potential in supercapacitors. Yang’s group [35] used banana peel as a carbon source to synthesize MnO_2_/biomass-based porous carbon (BPC) composites by the hydrothermal method. The MnO_2_/BPC composites electrode has a great specific capacitance of 139.6 F g^−1^ at the current density of 300 mA g^−1^ and 70 F g^−1^ at 10 A g^−1^. In addition, it loses 7.7% of the specific capacitance after 1000 cycles. In Shen’s research [124], soybean pod was used as the precursor of carbon matrix to prepare MnO_2_/soybean pod carbon (SPC) composites by a one-step in situ hydrothermal method. The prepared material retains the original tubular structure of the pods, with a large number of δ-MnO_2_ nanorods covered on the surface. According to the research, the MnO_2_/SPC electrode has a better specific capacitance than the MnO_2_ electrode (530 F g^−^^1^ and 362 F g^−^^1^, respectively). Besides, the symmetric MnO_2_/SPC-based supercapacitor displays high capacitance retention of 91% after 6000 cycles and maintains the energy density of 35.1 W h kg^−^^1^ at a high power density of 9000 W kg^−^^1^. The hollow structure of MnO_2_/carbon spheres (CSs) [125] was prepared by a simple one-step hydrothermal method. First, 60 mg hollow carbon was dispersed in 30 mL 20 mM KMnO_4_ aqueous solution by sonication. Then, this aqueous solution was heated at 160 °C for 3 h in a Teflon-lined stainless steel autoclave. After the reaction and cooling down, the product was vacuum filtered, washed, thoroughly dried at 60 °C, and then labeled as MnO_2_/hollow CS-20. An asymmetrical supercapacitor is assembled with the MnO_2_/hollow CS-30 material as the positive electrode and the hollow CS as the negative electrode. At the 2.0 V voltage window, the maximum energy density (41.4 W h kg^−1^) is obtained at a power density of 500 W kg^−1^. Moreover, the asymmetric supercapacitor shows a good cycling stability of 93.9% capacitance retention after 5000 cycles.

#### 2.3.2. Introduction of Battery-Type Metal Oxides

According to Nie’s study [126], introducing battery-type metal oxides into MnO_2_-based electrodes has been thought to be an effective way to increase the capacity and energy density of supercapacitors. Rapid redox reactions and synergistic effects of MnO_2_ and battery metal oxides improve the overall performance of the electrode. The electrode [127] obtained by depositing MnO_2_ on NiO nanosheets for 30 min has an ultra-high specific capacitance of 1227.2 F g^−1^ (at 10 A g^−1^) and a capacitance retention rate of 76.7% after 10,000 cycles. Via a low temperature facile chemical bath deposition, Liu et al. [128] fabricated leaf-like Co_3_O_4_@MnO_2_ nanosheets on the carbon cloth fibers (Co_3_O_4_@MnO_2_CC) with a hierarchical core–shell structure. The leaf-like Co_3_O_4_@MnO_2_ nanosheets exhibit brilliant electrochemical performance, including super-high specific capacitance (616.7 F g^−1^ at 2 A g^−1^) and superior cyclic stability (83.1% retention after 10,000 cycles at 20 A g^−1^) as well as high output potential of 1.2 V. The asymmetric supercapacitor (Co_3_O_4_@MnO_2_CC-90 as the cathode and AC as the anode) has energy density of 54.71 W h kg^−^^1^ at the power density of 1.06 kW kg^−^^1^ and 86.3% retention of specific capacitance after 10,000 cycles at 10 A g^−^^1^.

### 2.4. ZnO

**Figure 6:**
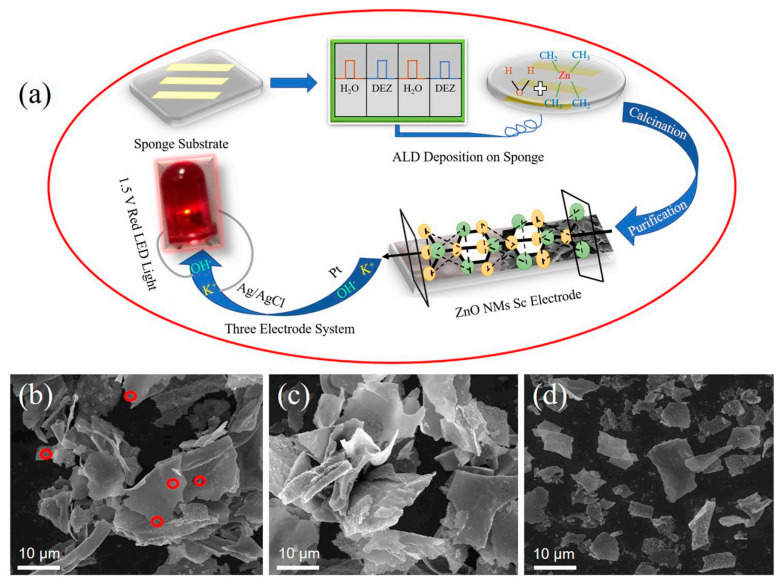
Design and morphologies of ZnO nanomembranes (NMs) with various thicknesses: (**a**) schematic of the fabrication procedure of ZnO NMs electrode and corresponding supercapacitor; (**b**–**d**) SEM images of ZnO NMs with (**b**) 50, (**c**) 100, and (**d**) 200 atomic layer deposition (ALD) cycles. The red circles in (**b**) represent the existence of the holes in ZnO NMs. Reproduced with permission from [134]. Copyright 2020 Elsevier.

Electrode materials largely determine the performance of supercapacitors [129]. Therefore, people have been looking for electrode materials with good electrochemical performance. TMOs have gotten extensive attention because of their high redox activity, good theoretical capacitance, and low costs [130]. ZnO is a well-known active material that has the characteristics of environmental protection, natural abundance, and ideal capacitance [50,131,132]. Via the chemical coprecipitation method, Dhivya Angelin et al. [133] modified the ZnO nanostructure with doping Zr. The experimental results indicate that 9 wt.% Zr-doped ZnO nanostructure has the best electrochemical performance of excellent specific capacitance of 518 F g^−1^ (at 1 A g^−1^) and capacitance retention of 94% (5000 successive galvanostatic charge-discharge cycles). ZnO nanomembranes (NMs) [134] were prepared with 100 atomic layer deposition (ALD) cycles. Figure 6a shows the fabrication procedure of ZnO NMs electrode and the corresponding supercapacitor. Figure 6b–d display the SEM images of the ZnO NMs with 50, 100, and 200 ALD cycles.

In different electrolytes, the ZnO NMs have different specific capacitance, 846 F g^−1^ (6 M KOH at 1 A g^−1^), 465 F g^−1^ (1 M KCl at 1 A g^−1^), and 65 F g^−1^ (6 M Na_2_SO_4_ at 1 A g^−1^), respectively. However, those materials mentioned all have the problem of capacity attenuation during long-term cycling [135,136].

#### 2.4.1. ZnO-Based Composite Electrode

Numerous ZnO-based electrode materials are fabricated, such as carbon material-ZnO electrode [137,138,139], medal oxides-ZnO electrode [140,141,142], and polymer-ZnO electrode [143,144], for the purpose of pursuing good electrochemical performance. With asphalt as carbon source and ZnO as template, graphene nanocapsules (GNCs) [145] were synthesized by in situ KOH activation technology. High-speed channels for electron conduction, micropores for ion adsorption, and short pores for ion transport are provided by the 3D porous network. As supercapacitor electrodes, the GNCs exhibit great capacitance of 194 F g^−1^ at 20 A g^−1^ (277 F g^−1^ at 0.05 A g^−1^) and capacitance loss of 2.6% after 15,000 cycles. Chebrolu et al. [146] fabricated a series of metal oxide composite nanostructures using the chemical bath deposition method. Those metal oxide composite nanostructures include ZnO nanowires, NiO nanosheets, ZnO/CuO nanowire arrays, ZnO/FeO nanocrystals, ZnO/NiO nanosheets, and ZnO/PbO nanotubes, among which ZnO/NiO nanosheets have the best electrochemical performance (at 8 mA cm^−2^ with a specific capacitance of 1248 F g^−1^ and long-term cycling stability). Because of ZnO/NiO nanosheets’ unique surface morphology, increasing the electron transfer rate and electrical conductivity, the energy storage properties are improved. Di’s group [147] reported that combing ZnO with a small amount of Al_2_O_3_ could prevent the ZnO nano-framework from collapsing. Meanwhile, the specific surface area of the material could be increased to enlarge the contact area between the composite and the electrolyte solution. The Al_2_O_3_-ZnO nanorod displays a high specific capacity (463.7 F g^−1^) and favorable cyclic stability (96.9%), which is higher than Al_2_O_3_ and ZnO. In addition, the ion transferred number and the ion diffusion coefficient of the Al_2_O_3_-ZnO nanorod are 6.3 and 7.6 × 10^−13^ cm^2^ s^−1^. Studies have shown that the electrochemical properties can be further improved by synthesizing the ZnO-based multicomponent electrodes [65,148,149,150]. Obodo et al. [151] synthesized Co_3_O_4_-CuO-ZnO@GO nanocomposite films by the hydrothermal method and then examined the effect of carbon ion irradiation on the properties of the prepared nanocomposite. The results of X-ray diffraction (XRD) suggest that low doses of carbon ion irradiation enhance the crystallinity of the materials, and high doses irradiation lead to deficiencies and disorder to the Co_3_O_4_-CuO-ZnO@GO, resulting in distortion and defects in the structure of the material. According to the study of electrochemical performance, the highest specific capacitance (1950 F g^−1^ at 10 mV s^−1^) is obtained at a radiation dose of 5.0 × 10^15^ ions cm^−2^, while the specific capacitance of 208 F g^−1^ and 1356 F g^−1^ are obtained at 1.0 × 10^15^ ions cm^−2^ and 7.0 × 10^15^ ions cm^−2^, respectively. Self-assembled ZnO-CoO@nitrogen-doped carbon (NC) electrode [152] was prepared by the hydrothermal method combined with annealing. Owing to high conductivity, rich oxygen vacancies, and mesoporous structure, the electrode based on ZnO-CoO@NC shows a good cycle stability of 92% specific capacitance retention at 2 A g^−1^ (40,000 cycles) without morphology change. Besides, the graphene//ZnO-CoO@NC device also has 94% capacitance retention after 10,000 cycles at 2 A g^−1^. At a potential window of 1.6 V, the device possesses an energy density of 16.5 W h kg^−1^ at a power density of 396.51 W kg^−1^ and remains at 5.5 W h kg^−1^ at 5634.5 W kg^−1^.

#### 2.4.2. Problems of ZnO-Based Electrode

A study [153] pointed out that the surface morphology of the powder coating became smoother and the exposed surface area lessened after a series of electrochemical reactions, because of which specific capacitance decreased. To solve this problem, ZnO rods are prepared by a hydrothermal method and then coated with a MoO_4_^2−^ modified carbon layer (Mo-C) [154]. The carbon coating is composed of many tiny carbon spheres of 10–50 nm in size. Owing to the mesoporous structure, ZnO@Mo-C possesses a larger specific surface area (96.36 m^2^ g^−1^) than pure zinc oxide (0.835 m^2^ g^−1^). In addition, compared with the electrode material prepared by Mohamed’s group [155], ZnO@Mo-C displays better capacitance and rate capability (900 F g^−1^ at 1 A g^−1^ and 650 F g^−1^ at 10 A g^−1^). He and co-workers [156] constructed ZnO flower nanostructures on nickel foam coated with hierarchical α-Co(OH)_2_. The unique structure increases the charge and ion transfer rates and improves the contribution of pseudoactive substances in faradaic reactions. An asymmetric supercapacitor is assembled in 3 M NaOH with NF@ZnO@Co(OH)_2_ as positive electrode and NF@cellulose paper as the negative electrode. In cell voltage of 1.5 V, the asymmetric supercapacitor exhibits maximum energy density of 62.57 W h kg^−1^ at a power density of 71 1.28 W kg^−1^ and, at 16,985.55 W kg^−1^ (maximum power density), it delivers an energy density of 16.04 W h kg^−1^. Ding et al. [157] anchored binder-free tin oxide nanosheets on nickel foam by a one-step hydrothermal method, and then CoS was deposited on the ZnO surface by electrodeposition. At a current density of 3 mA cm^−2^, ZnO@CoS shows a high specific capacity of 898.9 C g^−1^, while specific capacity of pristine ZnO and ZnO precursor are 614.4 C g^−1^ and 530.6 C g^−1^, respectively. The assembled button-type asymmetric supercapacitor displays an energy density of 45.2 W h kg^−1^ at 1039.1 W kg^−1^ with an areal capacity of 2438 mC cm^−2^. In addition, the capacity retention of 107% after 10,000 cycles shows its remarkable cycling stability. Table 1 shows the electrochemical performance of representative binary TMOs electrode materials.

### 2.5. XCo_2_O_4_ (X = Mn, Cu, Ni)

Ternary transition metal oxide XCo_2_O_4_ has a spinel structure. Generally, the two metal ions exhibit high capacity and conductivity, so ternary transition metal oxide has more abundant redox reactions and higher electrochemical activity than single metal oxides [158]. Thus, it has achieved great attention so far. When XCo_2_O_4_ is used as electrode material, the corresponding equations can be described as follows [52,159]:

(3) $${\mathrm{MCo}}_{2}O_{4}+H_{2}O+{\mathrm{OH}}^{-}↔\mathrm{MOOH}+2\mathrm{CoOOH}+e^{-}$$

(4) $$2\mathrm{CoOOH}+{\mathrm{OH}}^{-}↔2{\mathrm{CoO}}_{2}+H_{2}O+e^{-}$$

**Figure 7:**
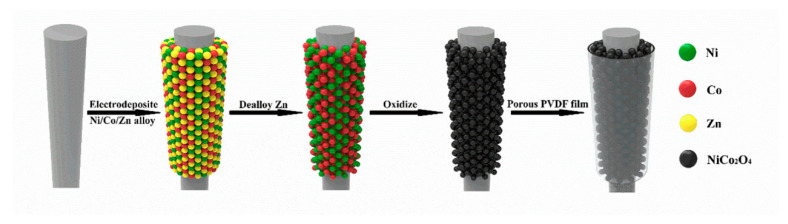
Schematic process showing the synthesis of porous polyvinylidene fluoride (PVDF)/NiCo_2_O_4_ coated on ultrafine nickel wire by electrodeposition and dealloying. Reproduced with permission from [162]. Copyright 2018 Elsevier.

Shanmugavadivel and co-workers [160] prepared a MnCo_2_O_4_ electrode by a simple solution combustion method. At the scanning rate of 10 mV s^−1^, the MnCo_2_O_4_ electrode shows the maximum specific capacitance of 270 F g^−1^, and the capacitance attenuation after 1000 cycles is 7.6%. MnCo_2_O_4_ nanosheets [161] prepared by galvanostatic electrodeposition have the maximum specific capacitance of 585 F g^−1^ at 0.2 mA cm^−2^. Furthermore, after 1500 cycles, the MnCo_2_O_4_ electrode possesses a capacity retention rate of 51.8%. A battery-type flexible electrode [162] was fabricated through coating mesoporous NiCo_2_O_4_ on an ultrafine nickel wire by electrodeposition, dealloying, and oxidation (Figure 7). The electrode is flexible and displays a high specific capacity of 315.4 C g^−1^ at 1 A g^−1^ and 8.4% area specific capacity loss after 50,000 cycles.

Via electrodeposition followed by air annealing, Pawar’s group [54] synthesized ultrathin nanoporous CuCo_2_O_4_ nanosheets on a nickel foam. After 5000 cycles, CuCo_2_O_4_ nanosheets show the specific capacitance of 1473 F g^−1^ at 1 A g^−1^ with the capacity retention rate of 93%.

#### 2.5.1. Core–Shell Structure for MCo_2_O_4_-Based Electrode

The electrochemical performance of the MCo_2_O_4_-based electrodes above is not ideal and does not meet the requirements of high-performance supercapacitors. Because of the lack of active sites, serious agglomeration, and destruction of electrode materials during the long cycle, the electrochemical performance of the electrodes is weakened [55]. The core–shell structure has a large number of electroactive sites and fast ion diffusion. Moreover, ions and electrons are accessible to the interior of the electroactive materials. So, the composites in the core–shell structure can possess a good cycle life and rate retention. At the same time, compared with a single component, the heterostructure composites with two constituents deliver better electrochemical performances owing to the synergistic effect [127,148]. Therefore, researchers speculate that the proper design of the core–shell structure can improve the cycle stability and prevent the collapse during the cycle [163].

Wang’s group [163] synthesized flower-like MnCo_2_O_4_ micro-nanostructures via a calcination process-assisted hydrothermal method. The prepared material possesses capacitance of 1933.33 F g^−1^ at 1 A g^−1^. The MnCo_2_O_4_@MnCo_2_S_4_//CNTs device delivers brilliant cycle stability of 96% capacitance retention after 5000 cycles. At the same time, it shows high energy density of 50.75 Wh kg^−1^ at 1260 W kg^−1^. Xu and co-workers [164] designed and prepared hierarchical mesoporous NiCo_2_O_4_@MnO_2_ core–shell nanowire arrays on nickel foam. After 8000 cycles at 50 mA cm^−2^, 113.6% original specific capacitance (1.23 F cm^−2^) was obtained. An asymmetric supercapacitor was assembled with the prepared sample as the positive electrode and AC as the negative electrode. The measurement results show that, with the potential window of 1.5 V, the asymmetric supercapacitor delivers a specific capacitance of 112 F g^−1^ at 1 mA cm^−2^ and a maximum energy density of 35 W h kg^−1^. In Zhu’s study [165], the unique 3D hierarchical structure of CuCo_2_O_4_@Ni(OH)_2_ provides large specific surface area, which enables rapid response and enhances electrochemical properties (2160 F g^−1^ at 1 A g^−1^ and a good capacitance retention of 82.7% at 20 A g^−1^).

#### 2.5.2. Introduction of Transition Metal Sulfides Materials

Transition metal sulfides have the characteristics of high faradic redox activities, unique physical or chemical properties, and long cycle performance. The conductivity of metal sulfides is higher than that of corresponding oxides, which is more convenient for electron transfer. In addition, low electroconductivity restricts TMOs further application in supercapacitors. At the same time, transition metal sulfides can make up for the shortcoming of TMOs. Moreover, because of the synergistic effect between the two individual materials, the mixed structure electrode can also improve the whole active region and promote the transport of ions from the electrolyte [19,166,167]. Liu and co-workers [167] fabricated Co_9_S_8_@NiCo_2_O_4_ nanobrushes by NiCo_2_O_4_ nanosheets grown on Co_9_S_8_ hollow nanoneedles. Owing to the good electrical conductivity and specific capacitance of Co_9_S_8_ and large specific surface area provided by the hollow nanoneedles structure, remarkable electrochemical properties are displayed (a high specific capacitance of 1966 F g^−1^ at 1 A g^−1^ and excellent capacitance retention of 92.9% after 5000 cycles). Owing to its unique structure, CuCo_2_S_4_/CuCo_2_O_4_ has the advantages of electrochemical surface area, excellent conductivity, short ion diffusion path, and rapid electron and ion transport rate [168]. Based on graphene aerogel and CuCo_2_S_4_/CuCo_2_O_4_, the asymmetric supercapacitor is synthesized, which delivers energy density of 33.2 W h kg^−1^, power density of 13.3 kW kg^−1^, and outstanding long-term stability of 73% capacitance retention after 10,000 cycles.

#### 2.5.3. Introduction of Oxygen Vacancies

**Figure 8:**
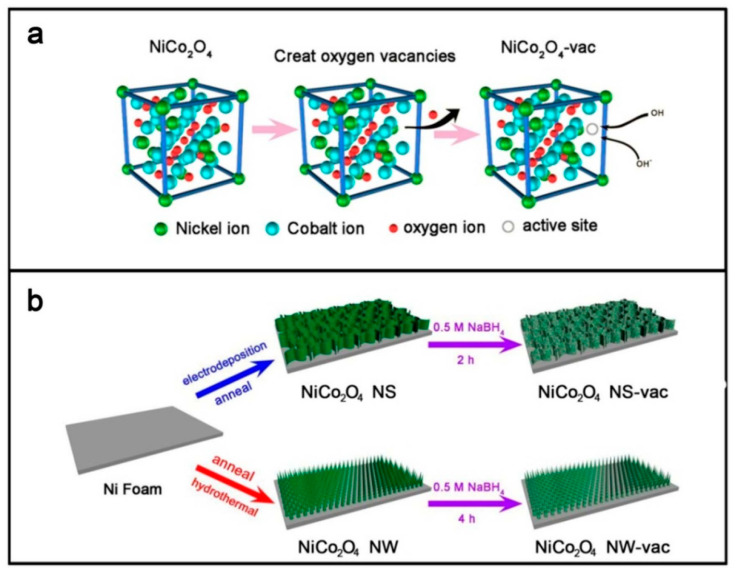
(**a**) Schematic diagram of oxygen vacancies in NiCo_2_O_4_; (**b**) the preparation scheme of the electrodes. Reproduced with permission from [171]. Copyright 2018 Elsevier.

There are three reasons that can explain why the introduction of oxygen vacancies is an effective strategy to improve the electrochemical performance. (i) Introduction of oxygen vacancies can adjust the surface chemical structure and enlarge the electroactive sur-face area exposed to the electrolyte. (ii) Oxygen vacancies can help the diffusion for charge carriers. Therefore, the conductivity of TMOs is improved. (iii) Electrochemical active sites are created when oxygen vacancies are introduced because the oxygen vacancies can serve as active sites for redox reactions. The kinetics of surface reactions is accelerated and the capacitance is increased [169,170,171]. To improve slow reaction kinetics and poor conductivity of CuCo_2_O_4_ electrode material, Feng et al. [170] prepared oxygen-vacancy-enriched CuCo_2_O_4_ nanoflowers in hypoxic atmosphere via a hydrothermal method followed by thermal treatment. The presence of oxygen defect sites and impurity bands provides massive reaction sites and fast ion intercalation, which improves electrical conductivity and hydrophilic properties. The electrode material shows a brilliant specific capacitance of 1.2 F cm^−2^ at 1.2 mA cm^−2^ and rate capability of 69.4% capacitance retention at 20 A g^−1^. Yan et al. [171] soaked the prepared NiCo_2_O_4_ nanostructures in the different concentration of NaBH_4_ solution (0.1 M, 0.3 M, 0.5 M, 0.7 M) to change the content of oxygen vacancies (Figure 8).

The results indicate that an appropriate oxygen vacancy content can increase the diffusion rate of carriers. In addition, oxygen vacancies as active sites for redox reactions can accelerate the surface kinetics reaction. The NiCo_2_O_4_ nanosheets electrode treated with 0.5 M NaBH_4_ has the best electrochemical performance (1590 F g^−1^ at 1 A g^−1^ and a capacitance retention of 79.12% at 10 A g^−1^ after 10,000 cycles).

### 2.6. AMoO_4_ (A = Co, Mn, Ni, Zn)

Compared with carbon materials, pseudocapacitive materials like metal oxides and organic conductive materials have higher specific capacitance owing to redox reaction on the surface of the material. In addition, ternary metal oxides have more active sites and faster redox reactions than single metal oxides, which become one of the materials to replace RuO_2_ [53,172,173,174]. As the hotspots of mixed transition metal oxides, the transition metal molybdates have attracted much attention because of their advantages including natural abundance, high theoretical specific capacitance, and low costs [175]. The performance of supercapacitors depends largely on the morphology and structure of their electrode materials. Therefore, it is very important to design electrode materials with unique spatial structure characteristics [176].

#### 2.6.1. Core–Shell Structure for AMoO_4_-Based Electrode

**Figure 9:**
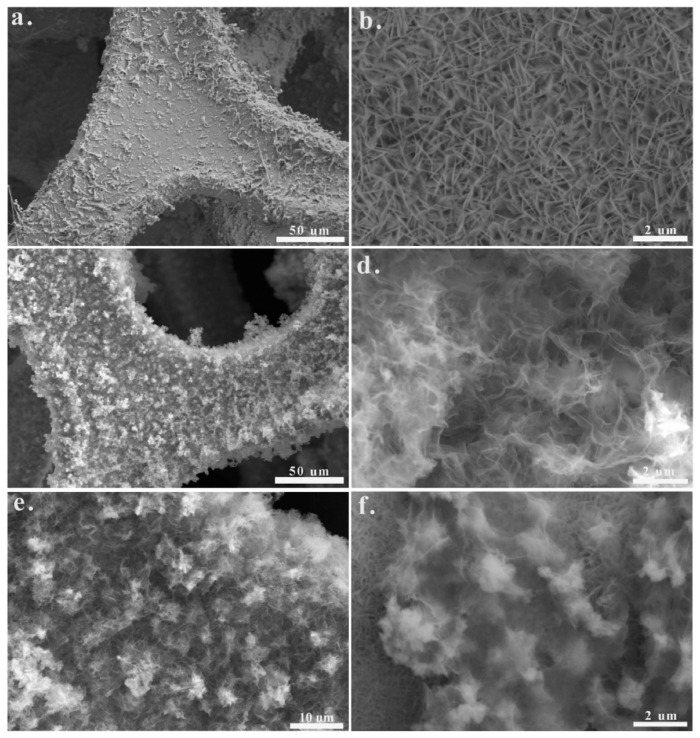
(**a**,**b**) SEM images of CoMoO_4_ with different magnification; (**c**,**d**) SEM images of CoS with different magnification; (**e**,**f**) SEM images of CoMoO_4_@CoS with different magnification. Reproduced with permission from [181]. Copyright 2020 Elsevier.

According to Yi’s study [127], the design of heterostructure composites has proved to be a hopeful way to promote the electrochemical properties of TMOs. Core–shell structure is a kind of heterostructure. The core–shell structure can supply large specific surface area with rich porosity. Meanwhile, the electron transfer rate is accelerated by core materials and electrochemical redox active sites are provided by shell materials. The volume changes during the cycle process are adjusted by the structure. In addition, the synergistic effect of each component is utilized to improve the electrochemical performance of the electrode [177,178,179,180]. Xuan et al. [181] designed and prepared 3D self-supported hierarchical CoMoO_4_@CoS core–shell heterostructures on rGO/Ni foam. Figure 9 shows the SEM images of CoMoO_4_, CoS, and CoMoO_4_@CoS with different magnification.

The unique material structure, the excellent conductivity of rGO, and the synergistic effect between the two materials help to increase the electrochemical active sites and enhance the capacitance. According to the electrochemical performance tests, the core–shell CoMoO_4_@CoS composite shows 81.1% retention of the initial capacitance (3380.3 F g^−1^ at 1 A g^−1^) after 6000 cycles at 10 A g^−1^. In order to further improve the performance of MCo_2_O_4_ materials, Hussain and co-workers [182] combined CoMoO_4_ with ternary metal sulfide NiCo_2_S_4_ to synthesize three-dimensional walking palm-like core–shell CoMoO_4_@NiCo_2_S_4_@NF nanostructure by a two-step hydrothermal method. At a current density of 5 mA cm^−2^, the synthesized composite exhibits a high capacitance of 17.0 F cm^−2^. Besides, 114% capacitance retention after 10,000 charge/discharge cycles reveals its remarkable cycling stability. The asymmetric supercapacitor fabricated with CoMoO_4_@NiCo_2_S_4_@NF and AC@NF as electrodes shows a high energy density of 60.2 W h kg^−1^ at a power density of 188 W kg^−1^. Moreover, it lights 22 parallel-connected red light emitting diodes for over 60 s.

#### 2.6.2. Modification of Quantum Dots

**Figure 10:**
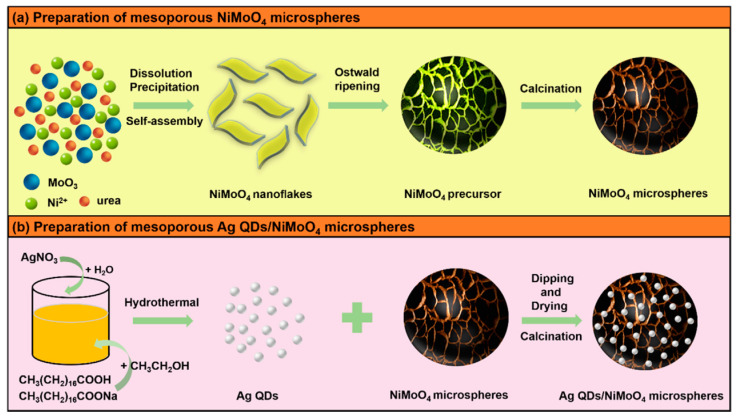
(**a**) Schematic illustration of the synthesis processes for mesoporous NiMoO_4_ microspheres; (**b**) schematic illustration of the synthesis processes for Ag quantum dots (QDs)/NiMoO_4_ microspheres. Reproduced with permission from [192]. Copyright 2020 Elsevier.

Quantum dots (QDs) are zero-dimensional nanoparticles of metal or semiconductor crystals with a diameter between 2 and 8 nanometers [183]. Quantum dots have attracted much attention for various applications owing to their characteristics such as quantum tunneling effect, surface effect, and quantum confinement effect [184]. It is noteworthy that the size of the material largely decides the substantial variation of fundamental electrical and optical properties [185]. Therefore, optical, electronic, and mechanical properties of QDs are different from corresponding nanoparticles [186]. For example, infinite exciton Bohr radius converts graphene to graphene quantum dots (GQDs) and GQDs start to fluoresce because electron distribution is transparently modified by the crystal boundary with the dimension of the crystal reducing to a nanometer scale [185]. Moreover, some unique properties of zero-dimensional nanoparticles like distinct edge and quantum confinement effects help to improve the electrochemical performance [183]. Some studies prove that quantum dot modification is an effective method for improving electrochemical performance [187,188,189]. Hence, it can be speculated that quantum dot modification is a promising strategy to improve the performance of AMoO_4_-based electrode. To the best of our knowledge, there are still relatively few reports in this regard. Zhu et al. [190] synthesized carbon quantum dots (CQDs) modified porous NiCo_2_O_4_ sphere composites. The CQDs/NiCo_2_O_4_ composite electrode possesses a specific capacitance (856 F g^−1^ at 1 A g^−1^) and excellent rate capability of 83.9%, 72.5%, and 60.8% capacity retention rate at 20, 50, and 100 A g^−1^, respectively. It also has 1.25% capacitance loss over 10,000 cycles at 5 A g^−1^, which indicates its exceptional cycling stability. The maximum capacitance of graphene quantum dot modified nano-needle electrode [191] at 1 A g^−1^ is four times higher than that of pure MnCo_2_O_4.5_ nano-spherical electrode (1625 F g^−1^ and 368 F g^−1^, respectively). In Zhang’s research [192], mesoporous NiMoO_4_ microspheres were modified by silver quantum dots to fabricate the Ag QDs/NiMoO_4_ microspheres (Figure 10).

**Figure 11:**
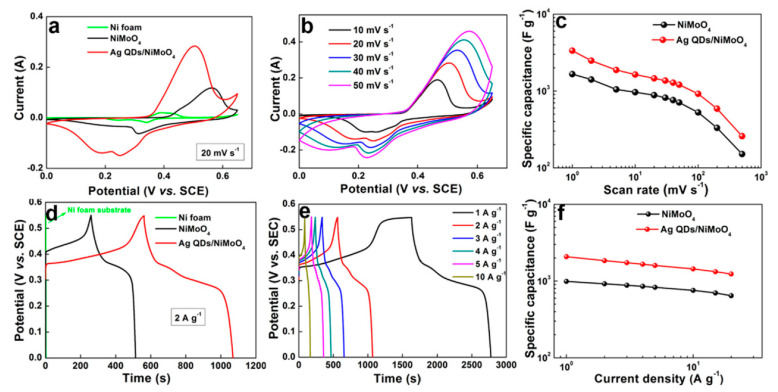
CV and GCD measurements for the NiMoO_4_ and Ag QDs/NiMoO_4_ electrodes in the three-electrode system: (**a**) contrastive CV curves at 20 mV s^−1^; (**b**) CV curves of Ag QDs/NiMoO_4_ electrode; (**c**) specific capacitances as a function of scan rates; (**d**) contrastive GCD curves at 2 A g^−1^; (**e**) rate performances of Ag QDs/NiMoO_4_ electrode; and (**f**) specific capacitances as a function of current densities. Reproduced with permission from [192]. Copyright 2020 Elsevier.

Owing to high specific surface area, plenty of active sites, enhanced interfacial conductivity, and porous structure, the Ag QDs/NiMoO_4_ microspheres exhibit specific capacitance of 3342.7 F g^−1^ at 1 mV s^−1^ and 2074 F g^−1^ at 1 A g^−1^ (Figure 11).

Moreover, the asymmetric supercapacitor assembled with Ag QDs/NiMoO_4_ and spore-derived activated carbon delivers high energy density of 48.5 W h kg^−1^ at a power density of 212.5 kW h kg^−1^ and good cycling performance. Table 2 shows the performance of representative electrodes based binary and ternary TMOs, and Table 3 shows the performance of representative supercapacitors based on binary and ternary TMOs.

## 3. Conclusions

The main purpose of developing supercapacitors is to meet the requirement of storing renewable energy. Supercapacitors have the advantages of high-power density, excellent cycle stability, and fast charge/discharge process. The performance of supercapacitors largely depends on the electrode materials. TMOs are the most favored because of their higher energy density and specific capacity than carbon materials, and better chemical stability than conductive polymers.

In this review, we have summarized recent developments of TMOs-based electrode materials for supercapacitors, including design, fabrication, and electrochemical performances. RuO_2_ is the ideal TMOs electrode material, but high cost and toxicity are its fatal shortcomings. Co_3_O_4_, MnO_2_, and ZnO are supposed to substitute RuO_2_. However, existing problems like low conductivity hinder their practical application in electrode materials. Therefore, we have introduced some strategies to solve the problems. (i) Preparing nano scale materials. The small size enlarges the contact surface and shortens the ion diffusion distance between materials and electrolyte. The size of material largely decides the substantial variation of fundamental electrical and optical properties. In addition, optical, electronic, and mechanical properties of QDs are different from corresponding nanoparticles. That is why QDs are very attractive in various fields. (ii) Utilizing the synergistic effect. It is very important that, through different combinations of carbon materials, transition metal oxides, transition metal sulfides, and other materials, the synergistic effect between different components can be used to improve the electrochemical performance effectively. In addition, some electrode materials containing metal sulfides exhibit good electrochemical performance, which indicates that other binary metal materials such as metal phosphides and metal nitrides also have potential for electrode materials. (iii) Synthesizing composites with a special structure. Porous structure, core–shell structure, and hollow structure can enlarge the surface area with generous electrochemical active sites and reduce the resistance of ion and electron transfer, thus increasing the conductivity and the rate of redox reaction. Further, the introduction of oxygen vacancies can not only increase active sites, but also adjust the surface chemical structure, improving the conductivity. (iv) Ternary transition oxides have higher conductivity, faster faradic reaction, and more active sites than single metal oxides. At the same time, Table 1, Table 2 and Table 3 show that ternary transition oxides have better performance than single transition oxides in the practical application of both electrode and supercapacitor. We believe that ternary TMOs will be the focus of future research on transition metal oxides.

Although much promising progress has been obtained in TMOs-based electrode materials, low energy density is still an obstacle for the development of supercapacitors. We have three recommendations on the development of supercapacitors. (i) Researchers should aim efforts towards how to obtain raw material of electrode with low cost and no pollution, and the use of biomass carbon materials is a good example. (ii) The development of portable and wearable electronic devices drives the study of flexible supercapacitors. That means, apart from increasing the energy density, it is necessary to achieve the stability of the capacitance performance under different bending and deformation conditions. (iii) At present, there are many reports on transparent and flexible electrodes for solar batteries. Moreover, supercapacitors based on battery-type electrodes are also widely reported. At the same time, hybrid supercapacitors can shorten the gap between conventional metal ion batteries and supercapacitors [52]. We wonder whether supercapacitors could have the possibility of solar charging in the future. If the wonder come true, that will represent significant progress in the development of supercapacitors.

## Figures and Tables

**Table 1:** The electrochemical performance of representative binary transition metal oxides (TMOs) electrode materials. SWCNT, single-walled carbon nanotube; CVD, chemical vapor deposition.

Material	Preparation Method	Electrolyte	Specific Capacitance	Cycling Stability	Reference
RuO_2_	hydrothermal method	1 M H_2_SO_4_	400 F g^−^^1^	84.7% (6000 cycles)	[56]
MoS_2_-RuO_2_	hydrothermal method	1 M KOH	972 F g^−^^1^	/	[59]
SWCNT/RuO_2_/PIn	oxidation polymerization of indole	1 M H_2_SO_4_	1307 F g^−^^1^	93% (3000 cycles)	[63]
reduced graphene oxide/Co_3_O_4_	hydrothermal method	2 M KOH	472 F g^−^^1^	95.6% (1000 cycles)	[83]
Co_3_O_4_ nanowires	hydrothermal method	3 M KOH	310.4 C g^−^^1^	80.3% (6000 cycles)	[87]
CeO_2_/Co_3_O_4_/rGO nanoparticles	hydrothermal method	6 M KOH	1606.6 F g^−^^1^	/	[92]
porous carbon/Co_3_O_4_	pyrolysis of precursor	3 M KOH	423 F g^−^^1^	17% decay 2000 cycles	[100]
MnO_2_ nanosheets/hollow carbon nanofibers	hydrothermal method	1 M Na_2_SO_4_	293.6 F g^−^^1^	/	[104]
N-doped carbon@MnO_2_	tannic acid-assisted etching process	1 M Na_2_SO_4_	247.9 F g^−^^1^	82.9% (2000 cycles)	[117]
graphene/MnO_2_ foam	CVD	1 M Na_2_SO_4_	333.4 F g^−^^1^	92.2% (2000 cycles)	[123]
nitrogen-doped porous hollow carbon spheres/MnO_2_	hydrothermal pre-carbonization and pyrolysis carbonization	1 M Na_2_SO_4_	255 F g^−^^1^	89% (5000 cycles)	[125]
Zr-doped ZnO	physio-chemical	1 M KOH	518 F g^−^^1^	94% (5000 cycles)	[133]
ZnO nanomembranes	atomic layer deposition	6 M KOH	846 F g^−^^1^	89% (5000 cycles)	[134]
ZnO@rGO	direct microwave irradiation	0.1 M KOH	102.4 F g^−^^1^	82.5% (3000 cycles)	[137]
Co_3_O_4_-CuO-ZnO/GO	hydrothermal method	0.5 M Na_2_SO_4_	2045 F g^−^^1^	83.02% (5000 cycles)	[151]

**Table 2:** The performance of electrodes based on binary and ternary TMOs.

Category	Electrode Material	Electrolyte	Specific Capacitance	Cycling Stability	Reference
binary transmission metal oxides	CuCo_2_O_4_/CuO@RuO_2_	2 M KOH	862.5 mAh cm^−^^2^	90.1% (8000 cycles)	[64]
	NiO/Co_3_O_4_	3 M KOH	1242 C g^−^^1^	95.5% (12,000 cycles)	[75]
	Co_3_O_4_ micro-bundles	2 M KOH	282.3 C g^−^^1^	74.6% (4000 cycles)	[51]
	Ag-doped Co_3_O_4_/NF	3 M KOH	1425 F g^−^^1^	96.4 (5000 cycles)	[91]
	Co_3_O_4_@MnO_2_ on carbon cloth	1 M Na_2_SO_4_	616.7 F g^−^^1^	83.1% (10,000 cycles)	[128]
	NiO@MnO_2_	/	1219.2 F g^−^^1^	76.7% (10,000 cycles)	[127]
	ZnO/CeO_2_	0.2 M K4(Fe[CN]_6_) in 3 M KOH	495.4 F g^−^^1^	95.6% (2000 cycles)	[140]
	ZnO@Mo–C	2 M KOH	900 F g^−^^1^	/	[154]
ternary transmission metal oxides	graphene quantum dots/MnCo_2_O_4.5_	2M KOH	1625 F g^−^^1^	80% (5000 cycles)	[191]
	Ag QDs/NiMoO_4_	3 M KOH	2074 F g^−^^1^	81% (1000 cycles)	[192]
	CoMoO_4_@NiCo_2_S_4_@Nickel Foam	3 M KOH	17.0 F cm^−^^2^	114% 10,000 cycles.	[182]
	CuCo_2_O_4_@Ni(OH)_2_	2 M KOH	2160 F g^−^^1^	92% (5000 cycles)	[165]
	Co_9_S_8_@NiCo_2_O_4_	3 M KOH	1966 F g^−^^1^	92.9% (5000 cycles)	[167]
	CoMoO_4_@MoZn_22_	3 M KOH	923 C g^−^^1^	92.3% (7000 cycles)	[177]

**Table 3:** The performance of supercapacitors based on binary and ternary TMOs.

Electrode Material	Operating Voltage	Specific Capacitance	Energy Density	Cycling Stability	Reference
RuO_2_//h-WO_3_	1.6 V	47.59 F g^−^^1^	16.92 W h kg^−^^1^	~171.75% (6500 cycles)	[72]
Co_3_O_4_//AC	1.6 V	310.4 C g^−^^1^	0.4 mW h cm^−^^3^	79.2% (10,000 cycles)	[87]
Co_3_O_4_-NiO/GO//AC	1.65 V	133 F g^−^^1^	50.2 W h kg^−^^1^	82% (3000 cycles)	[91]
Co_3_O_4_/NF—8 h//N-rGO/NF	1.6 V	62.5 F g^−^^1^	22.2 W h kg^−^^1^	93.3% (10,000 cycles)	[93]
MnO_2_/HCS-30//HCS	2.0 V	74.5 F g^−^^1^	41.4 W h kg^−^^1^	93.9% (5000 cycles)	[125]
Co_3_O_4_@MnO_2_@CC-90//AC	2.2 V	309.2 mF cm^−^^2^	54.71 W h kg^−^^1^	86.3% (10,000 cycles)	[128]
graphene electrode//ZnO-CoO@NC	1.6 V	/	16.5 W h kg^−^^1^	about 94% (10,000 cycles)	[152]
ZnO@CoS//AC	1.6 V	2438 mC cm^−^^2^	45.2 W h kg^−^^1^	107% (11,000 cycles)	[157]
NiCo_2_O_4_@MnO_2_//AC	1.5 V	112 F g^−^^1^	35 W h kg^−^^1^	~71% (5000 cycles)	[164]
CuCo_2_S_4_/CuCo_2_O_4_//graphene aerogel	1.6 V	90.4 F g^−^^1^	33.2 W h kg^−^^1^	73% (10,000 cycles)	[168]
CoMoO_4_@CoS//AC	1.7 V	189.5 F g^−^^1^	59.2 W h kg^−^^1^	91.5% (6000 cycles)	[181]
CoMoO_4_@NiCo_2_S_4_@NF// AC@NF	1.6 V	182 F g^−^^1^	60.2 W h kg^−^^1^	84% (5000 cycles)	[182]
MnCo_2_O_4.5_-40 GQDs//rGO	1.3 V	200 F g^−^^1^	46 W h kg^−^^1^	77% (5000 cycles)	[191]
Ag QDs/ NiMoO_4_//spore-derived AC	1.7 V	120.5 F g^−^^1^	48.5 W h kg^−^^1^	84.4% (5000 cycles)	[192]
